# A simple way to minimize cross infection from tear droplets during noncontact air-puff tonometry

**DOI:** 10.1017/ice.2020.1232

**Published:** 2020-09-28

**Authors:** Fen Tang, Chen Qin, Ningning Tang, Li Jiang, Fan Xu

**Affiliations:** 1 Department of Ophthalmology, People’s Hospital of Guangxi Zhuang Autonomous Region, Nanning, China; 2 Department of Ophthalmology, Renmin Hospital of Wuhan University, Wuhan, China

*To the Editor*—Noncontact air-puff tonometry (NCT) is a routine ophthalmological examination used to measure intraocular pressure (IOP). During NCT, tears might be scattered after the pulse of pressurized air on the cornea.^1^ It has been reported that severe acute respiratory coronavirus virus 2 (SARS-CoV-2) can be identified in the tears of patients with coronavirus disease 2019 (COVID-19).^2,3^ Considering the risk of cross infection through tear droplets, we investigated the contamination during NCT. In addition, we describe an effective protective measure to reduce cross infection.

The experimental procedure was performed using a volunteer with normal IOP and tear secretion. After applying 0.4% fluorescence sodium, the volunteer received the IOP measurement. Thereafter, the NCT machine was photographed to screen the droplets under a blue LED light. Next, we employed a protective measure and investigated its ability to prevent contamination from tear droplets. A transparent plastic shield was installed between the chin rest and forehead rest and the main mobile unit, and a homemade paper funnel was installed on the sensor (Fig. 1A). The IOP measurement and droplets screening were repeated using the same volunteer with the protective measure.

**Fig. 1.**
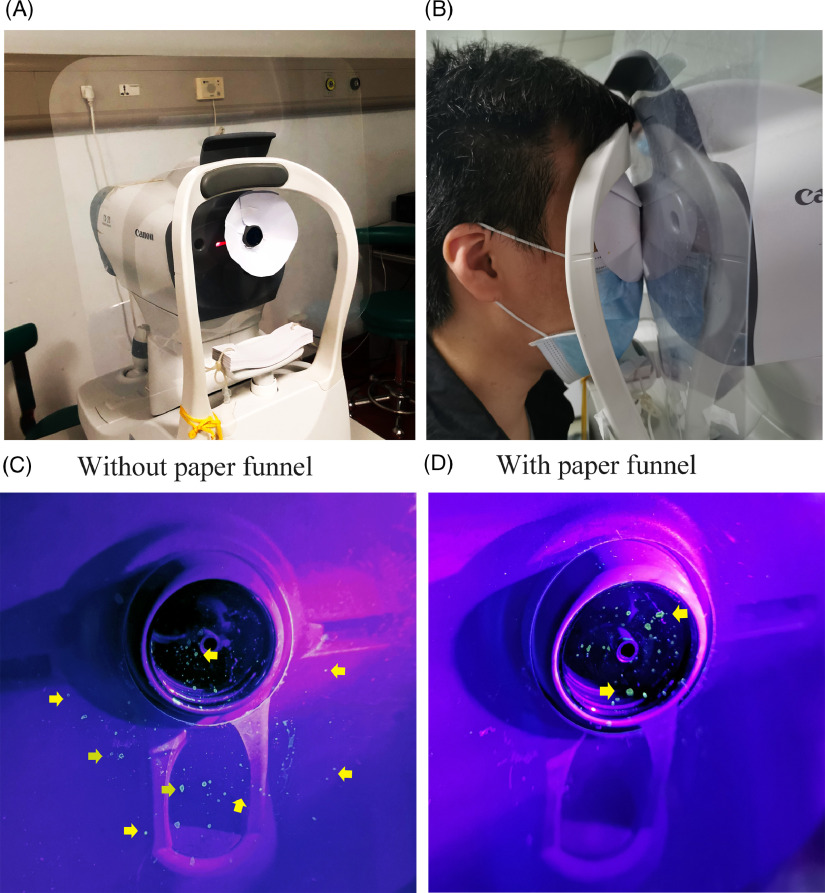
The screening of the scattered droplets with fluorescent dye on the NCT machine with the novel protective measure. (A) A transparent plastic shield (41 cm × 36 cm) and a homemade paper funnel were installed on the NCT machine. (B) The volunteer positioned his head in the chin and forehead rest, and his eye was covered by the funnel. (C) Scattered tear droplets on the shield and sensor with the plastic shield only (yellow arrow). (D) Scattered tear droplets on the sensor with the plastic shield and the paper funnel (yellow arrow).

Many scattered droplets were found on the NCT instrument after IOP measurement, including the main mobile main unit (Supplementary Fig. 1A–D online) and the sensor (Supplementary Fig. 1E online). We also detected some small droplets at the base of main unit and on the column of the chin and forehead rest (data not shown). The findings indicate that the NCT machine would have been contaminated widely if the IOP measurement was performed on an infected individual and that routine examinations posed a great risk for virus transmission from tears.

However, when we performed the IOP measurement with the shield, the droplets were dispersed on the shield instead of the main unit (Fig. 1C). Although the shield reduced the contamination of the main unit, it could not prevent the contamination of sensor, base, and column. Thus, a potential risk of cross infection remained, even with the shield.

Therefore, we designed and added a paper funnel to the sensor (Fig. 1A). The funnel could cover the examinee’s eye when performing the IOP measurement (Fig. 1B). As shown in Fig. 1D, droplets were only observed on the sensor, with no obvious dye found on the other parts of the machine.

We have demonstrated that tears could be dispersed during NCT in the absence of any protective barrier. Moreover, the scattered droplets were widely distributed on the surface of the tonometry equipment, even on the margin of the main unit and the column of chin rest, which would be touched by examiners frequently. Our findings reinforce the necessity of wearing protective equipment and of disinfecting tonometry equipment totally when performing NCT on people with infectious diseases such as COVID-19.

Considering the medical supply shortage and the difficulty of complete disinfection, we adopted an easy protective measure on NCT and demonstrated that it could reduce the number of infectious scattered droplets reaching the machine and could minimize the dispersal distance and area. With the shield only, droplet dispersion was limited to the shield and sensor. With the shield and paper funnel, droplet dispersion was limited to the sensor. Although such a barrier could not prevent the dispersion of tears completely, it would simplify the workflow of disinfection greatly. Additionally, the disposable paper funnel should be changed after each examination to avoid potential cross infection between patients.

Collectively, such a protective measure during air-puff tonometry should be recommended to minimize the risk of cross infection and to protect frontline ophthalmologists during the ongoing COVID-19 pandemic.

## Supplementary material

For supplementary material accompanying this paper visit http://dx.doi.org/10.1017/ice.2020.1232. click here to view supplementary material
