# Supplementary material for: A simple way to minimize cross infection from tear droplets during noncontact air-puff tonometry
Source: Infect Control Hosp Epidemiol. 2020 Sep 28;42(10):1296–7. doi: 10.1017/ice.2020.1232 (PMC7550885; doi:10.1017/ice.2020.1232)
Supplement: Supplementary file 1 [file S0899823X20012325sup.zip › S0899823X20012325sup002.docx]

**Supplementary Fig. 1.** The screening of the scattered droplets with fluorescent dye on NCT machine without any protective measure. (A) Scattered tear droplets with fluorescent dye on the main mobile unit (upper left). (B) Scattered tear droplets on the main mobile unit (upper right). (C) Scattered tear droplets on the main mobile unit (lower left). (D) Scattered tear droplets on the main mobile unit (lower right). (E) Scattered tear droplets on the sensor.
