# Supplementary figures and images for: A simple way to minimize cross infection from tear droplets during noncontact air-puff tonometry
Source: Infect Control Hosp Epidemiol. 2020 Sep 28;42(10):1296–7. doi: 10.1017/ice.2020.1232 (PMC7550885; doi:10.1017/ice.2020.1232)

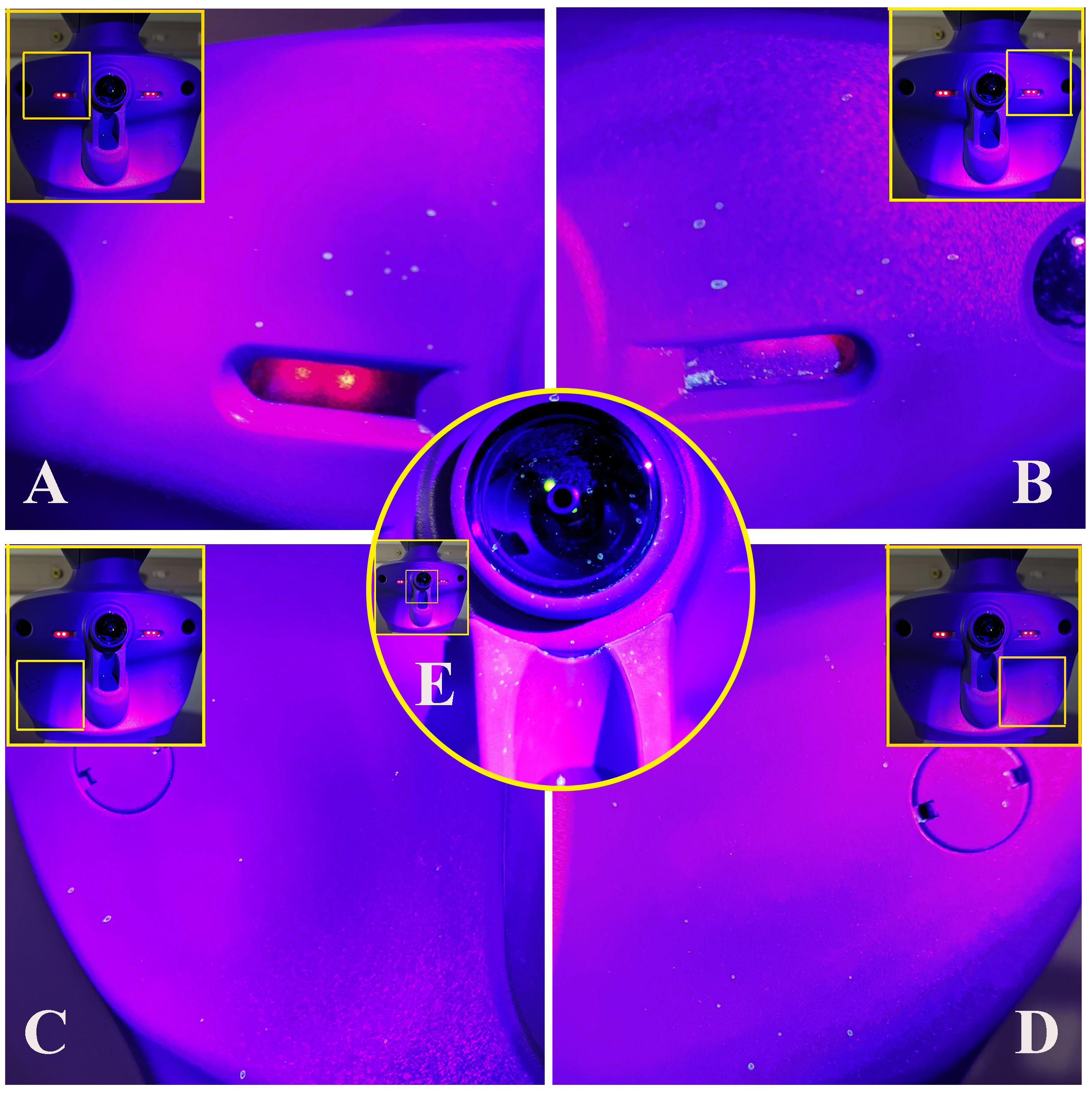

Supplement: Supplementary file 1 [file S0899823X20012325sup.zip › S0899823X20012325sup001.tif]
